# Supplementary figures and images for: Detection of TurboID fusion proteins by fluorescent streptavidin outcompetes antibody signals and visualises targets not accessible to antibodies
Source: eLife. 2024 Aug 29;13:RP95028. doi: 10.7554/eLife.95028 (PMC11361705; doi:10.7554/eLife.95028)

Source data 1. Uncropped and labelled gels for Figure 5—figure supplement 1.

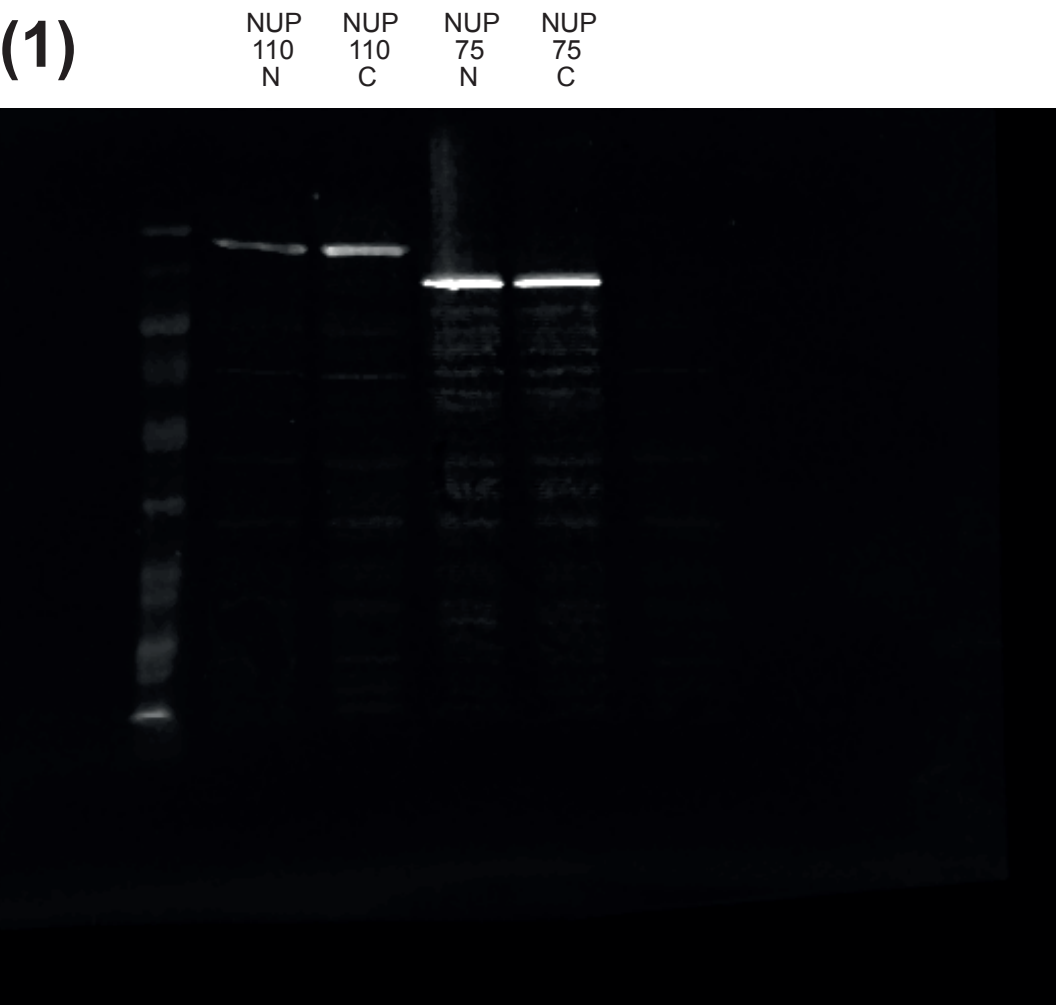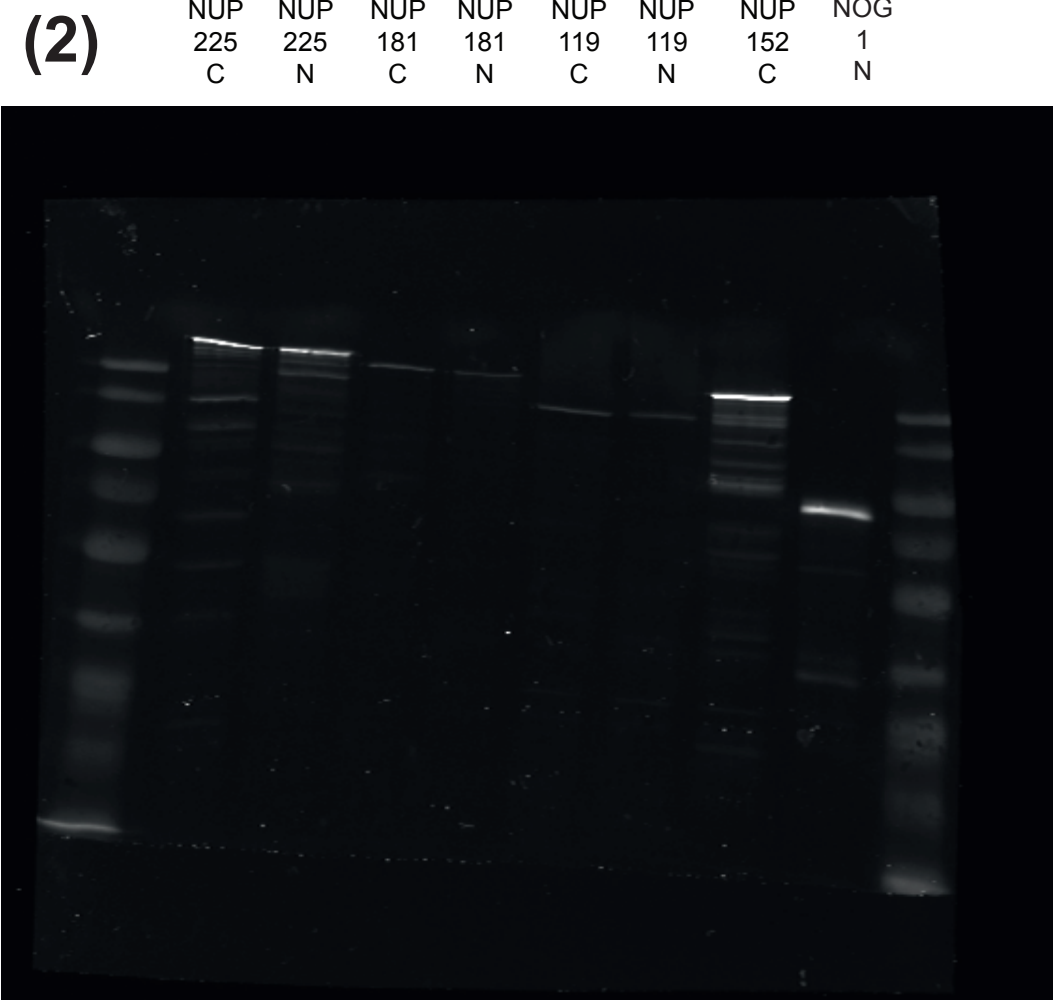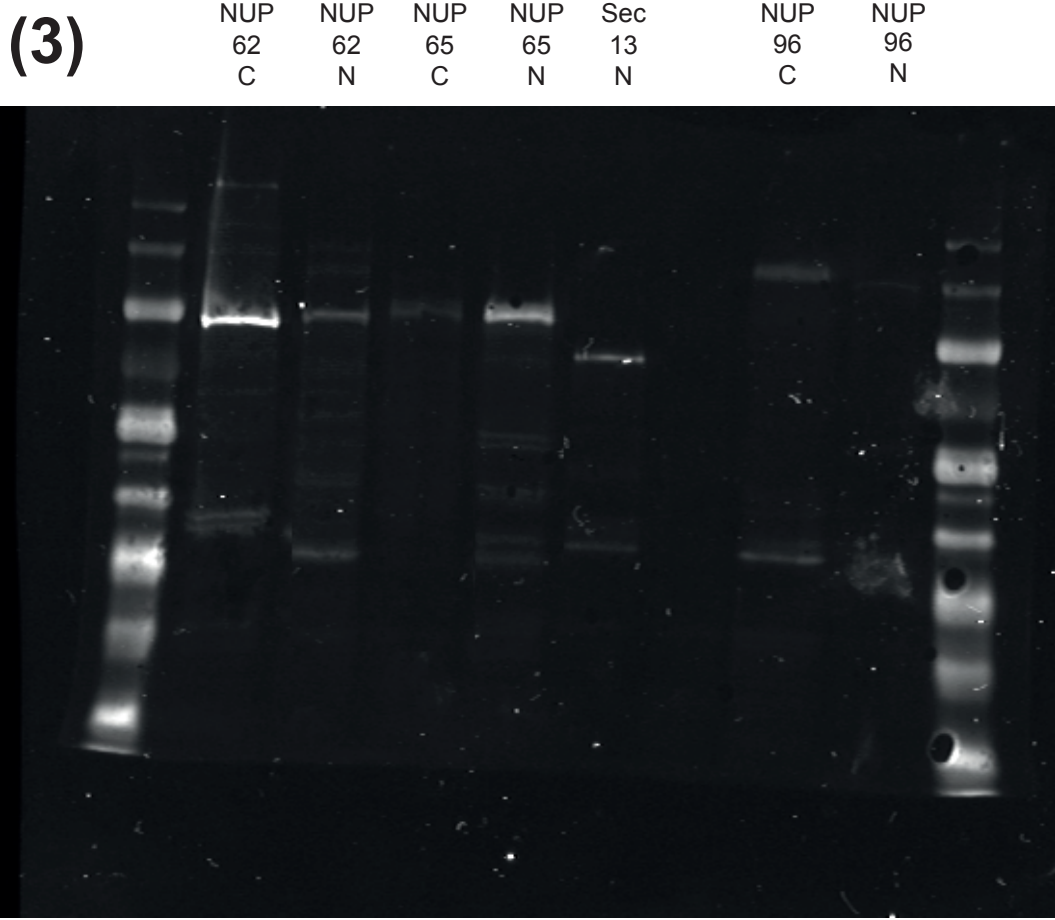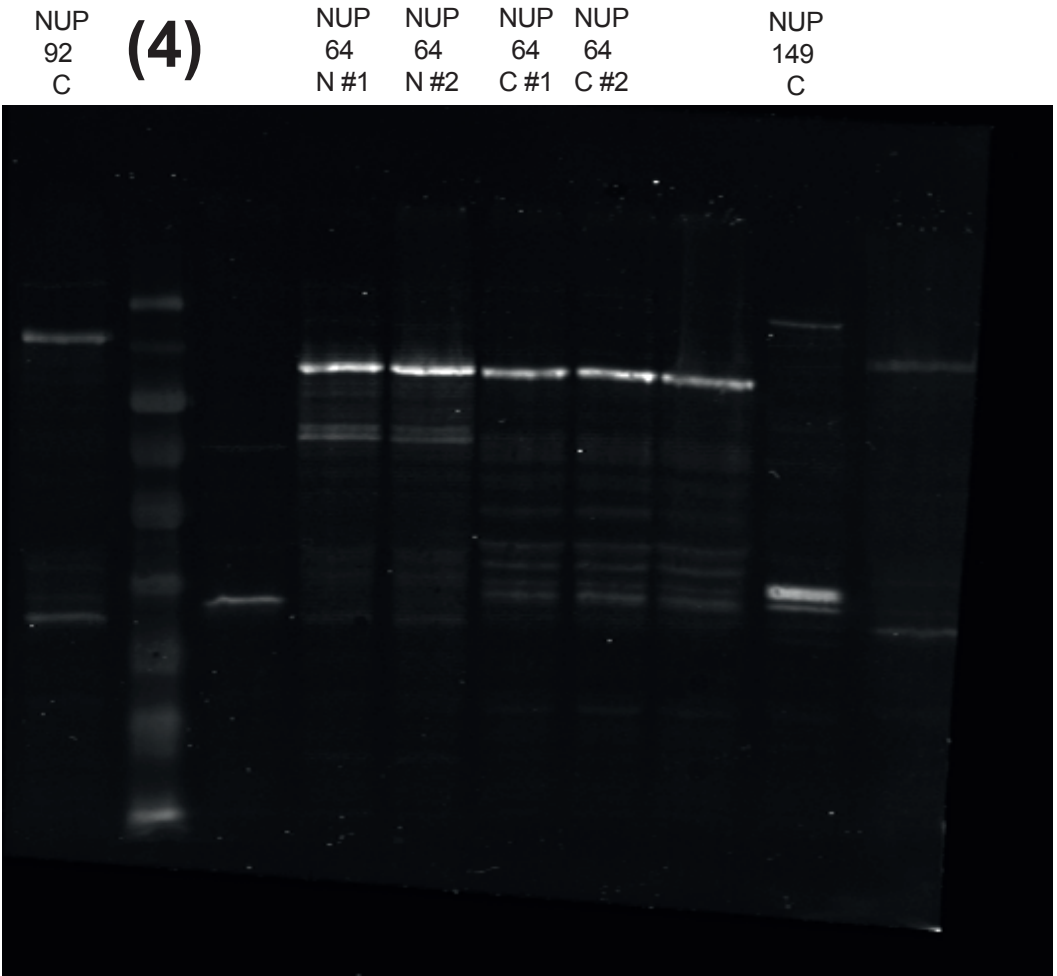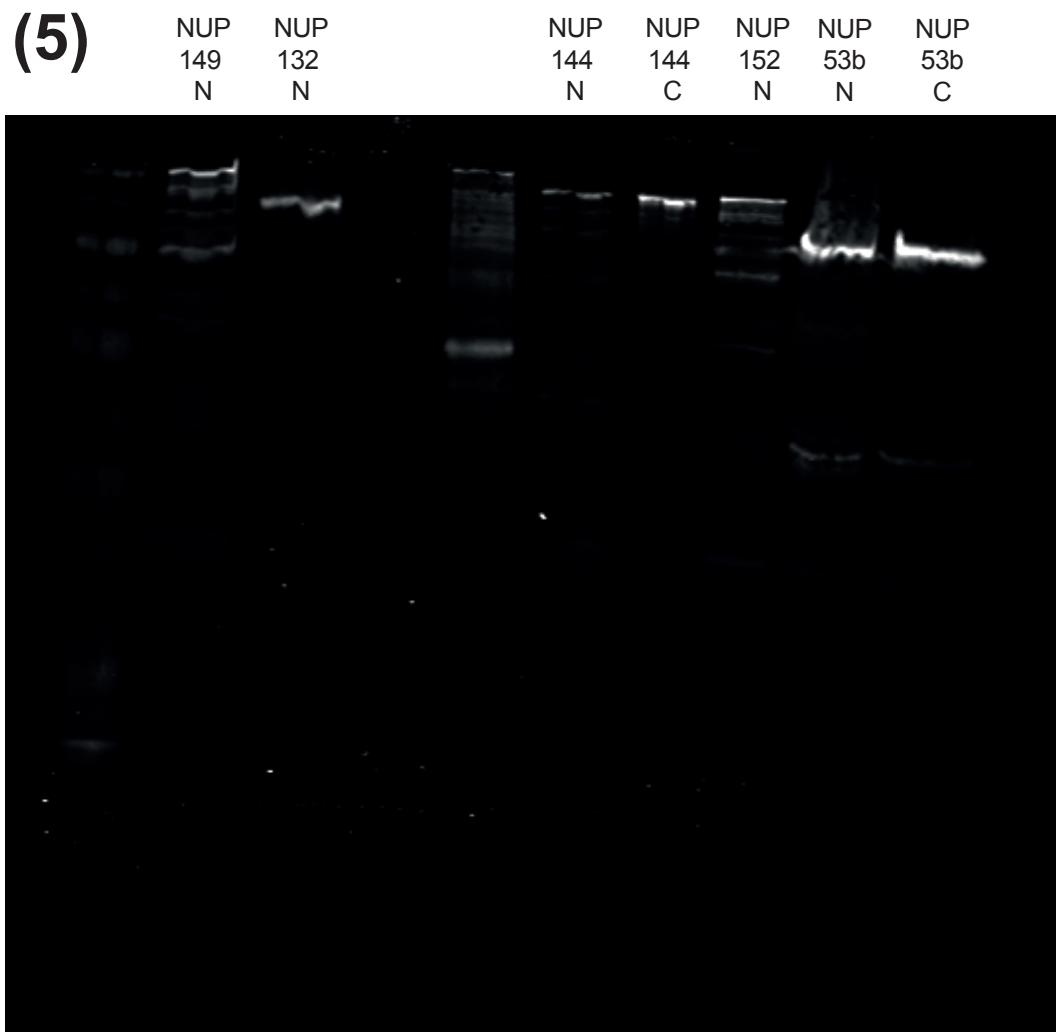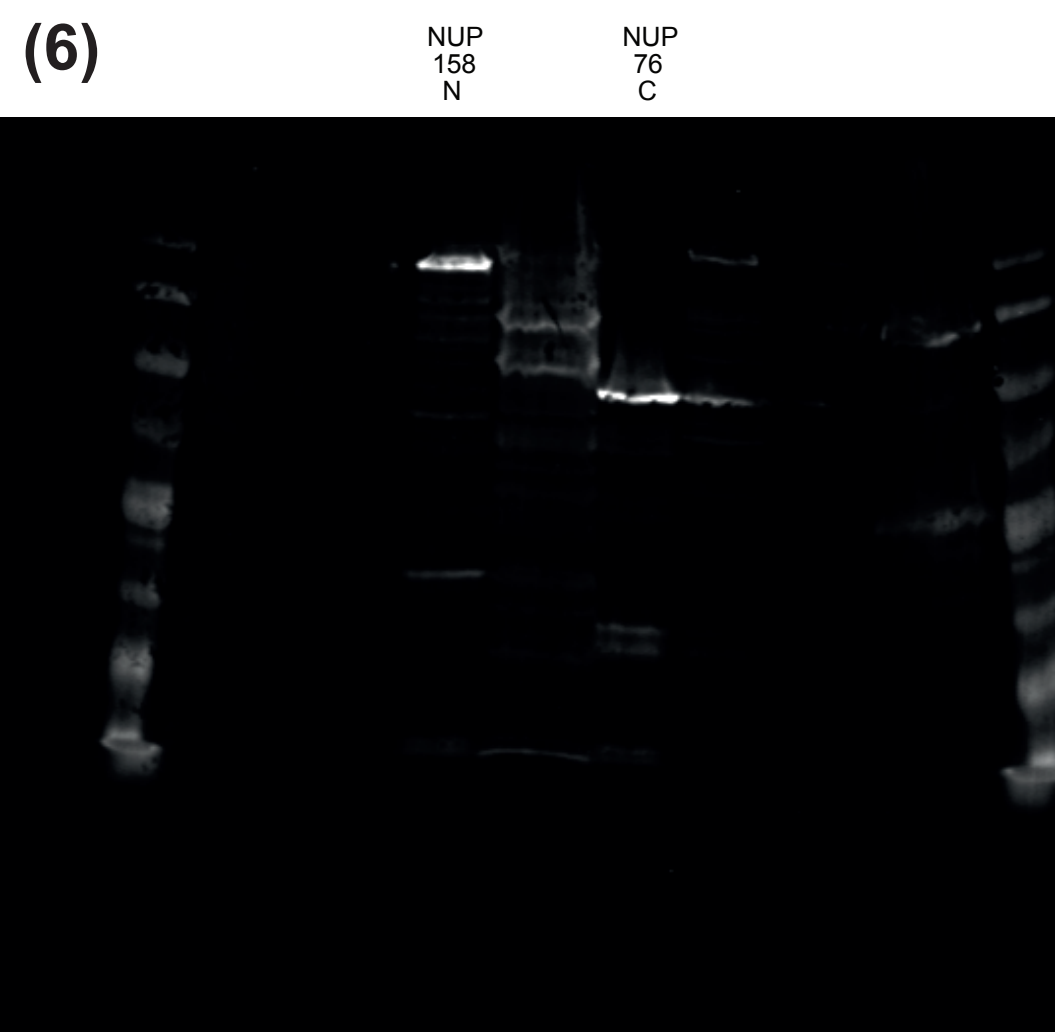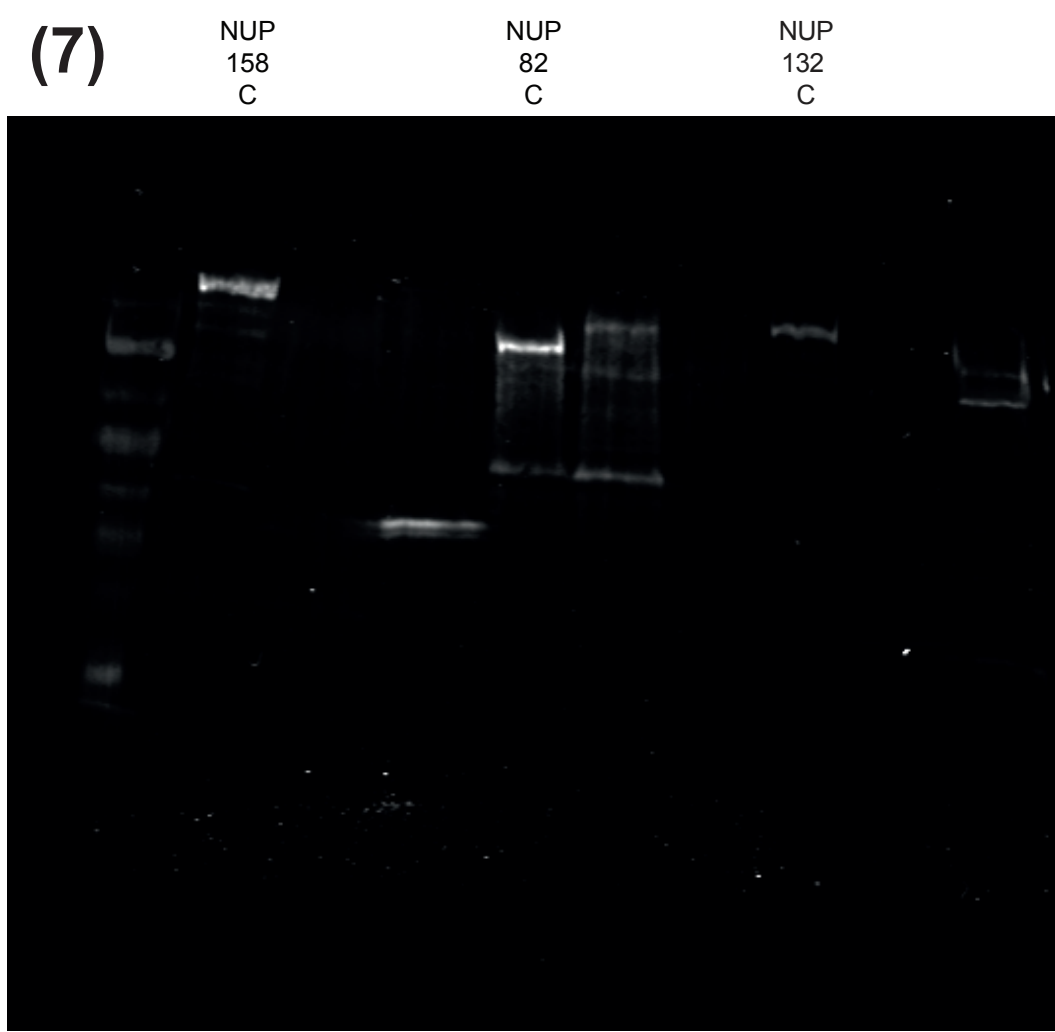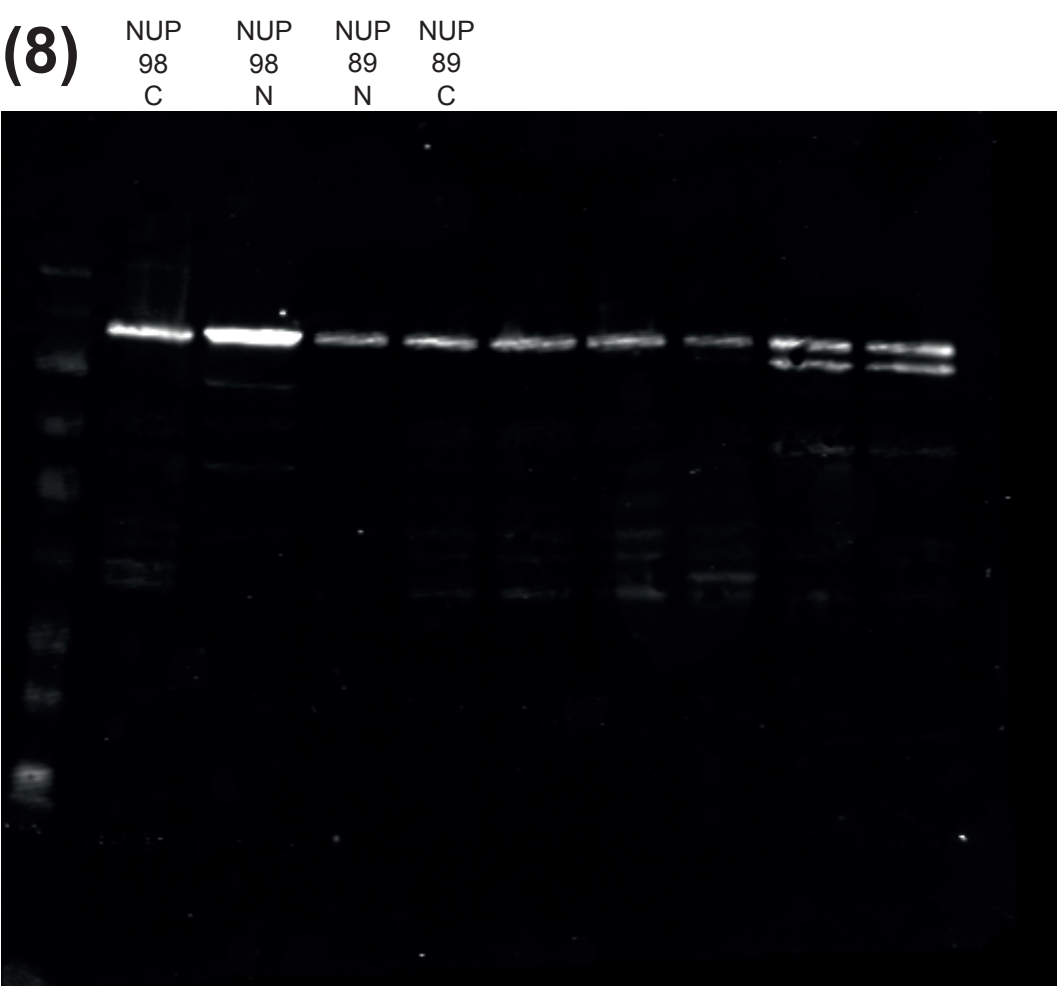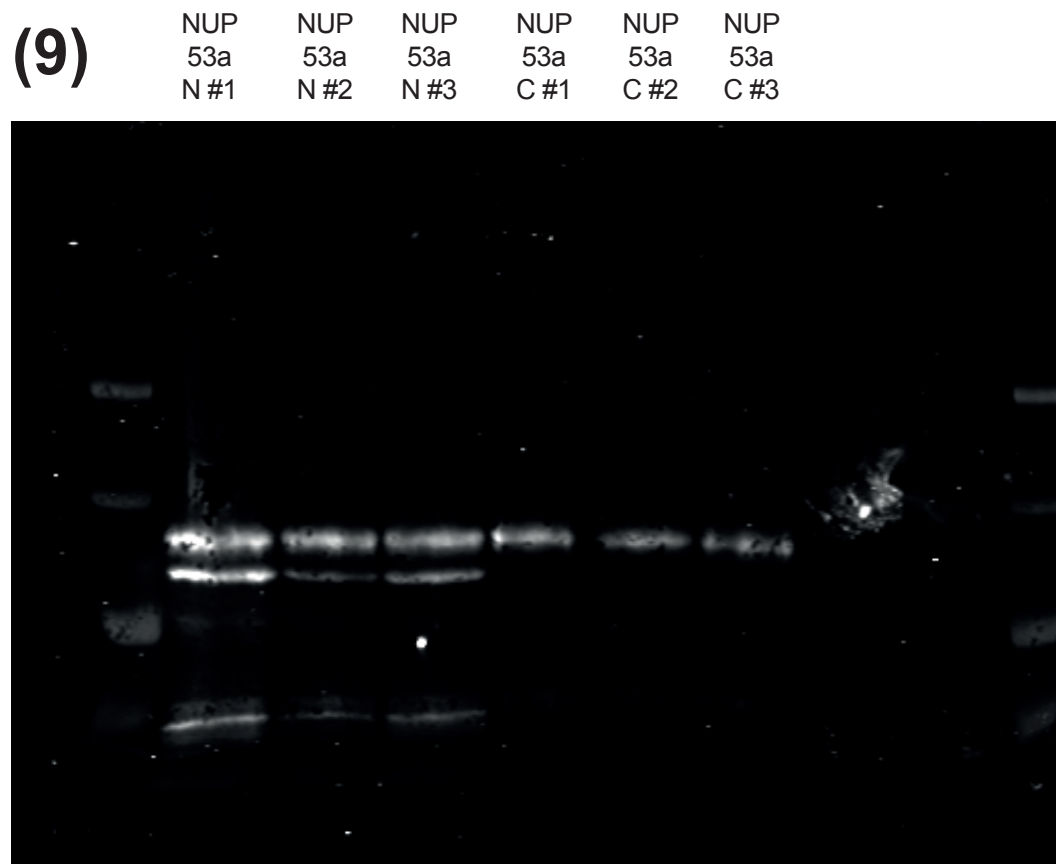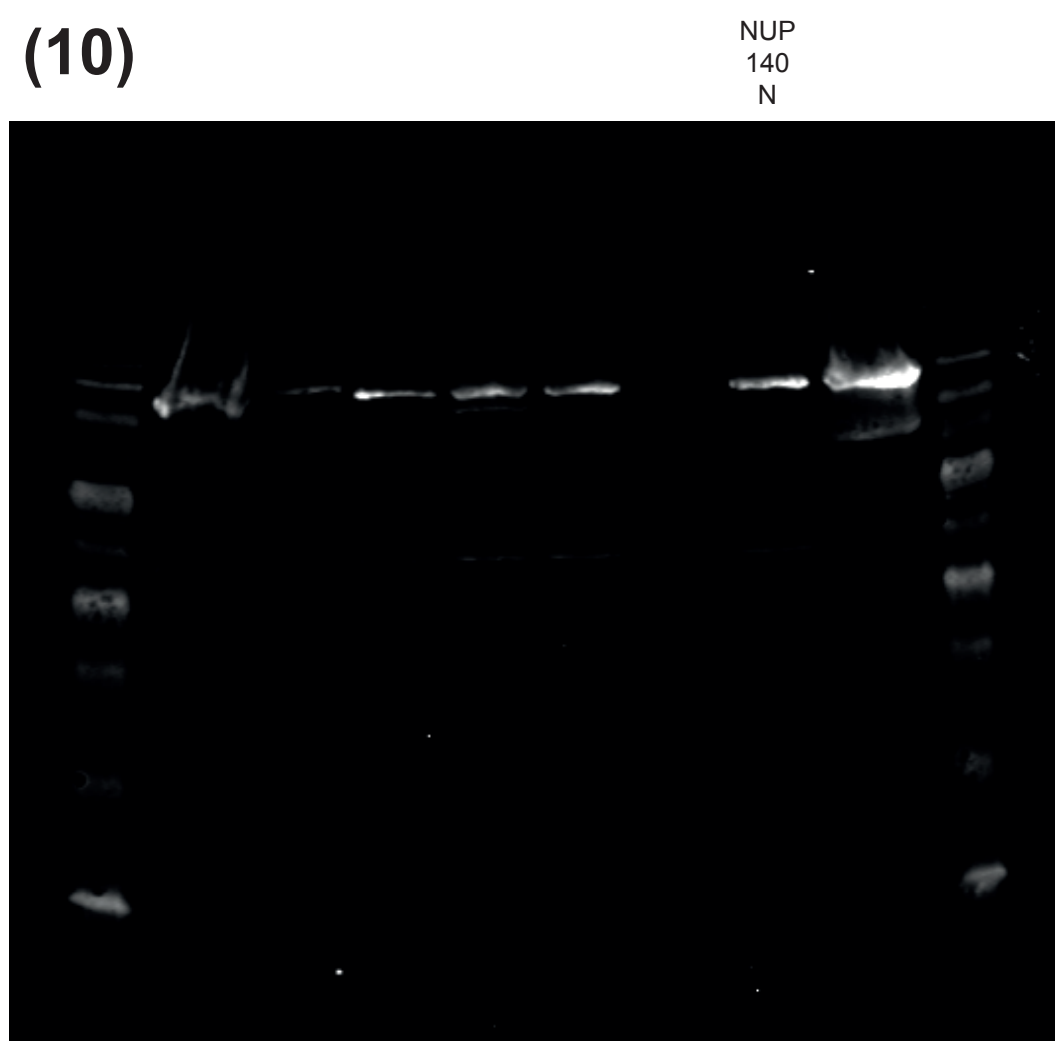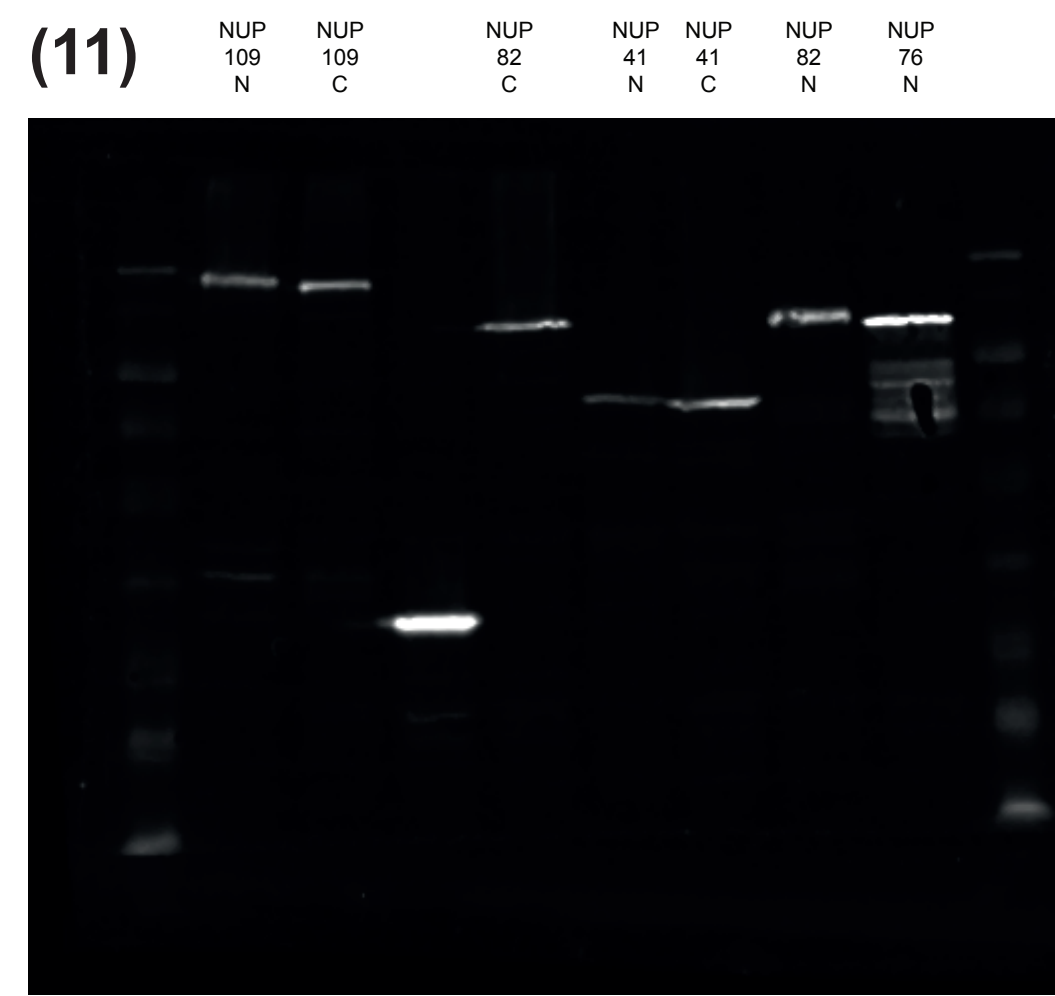

Supplement: Figure 5—figure supplement 1—source data 1. [file elife-95028-fig5-figsupp1-data1.pdf]

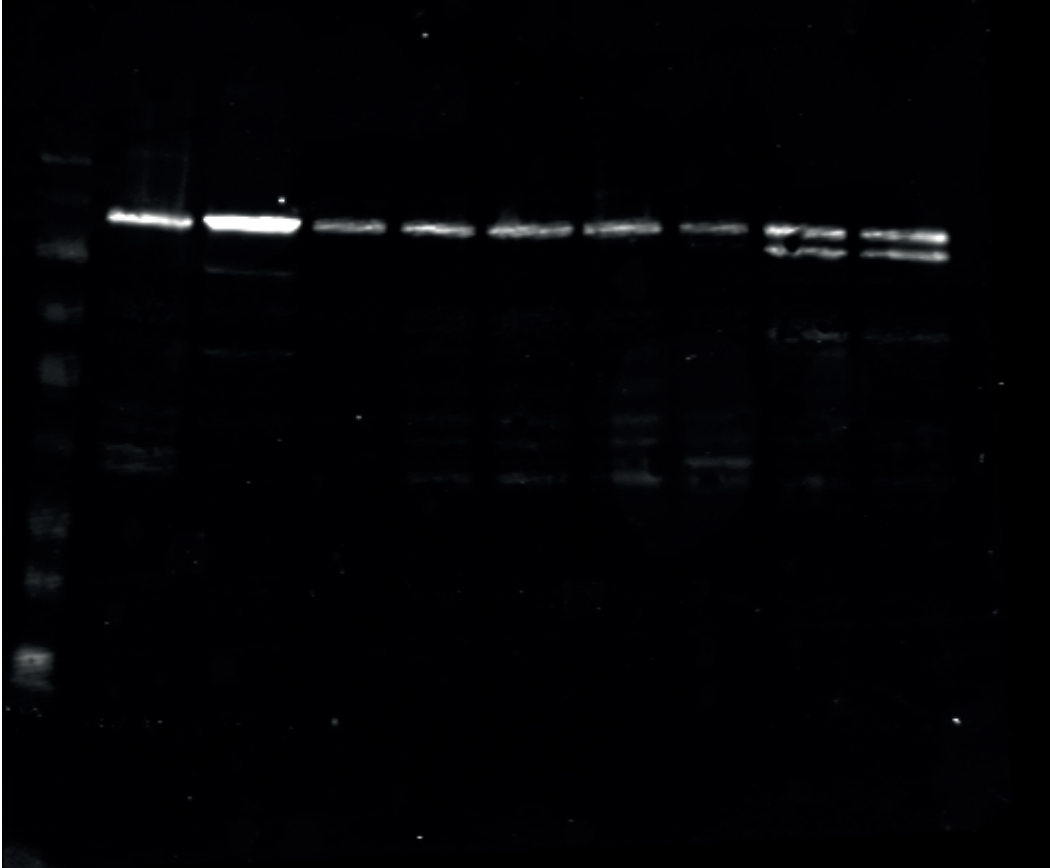

Supplement: Figure 5—figure supplement 1—source data 2. [file elife-95028-fig5-figsupp1-data2.zip › eLife.95028.SourceData2.Raw and unedited_gels_for_Figure5_FigureSupplement1/Figure 5ΓÇôfigure supplement 1-source data 2. Uncropped and Unlabeled gels for Figure 5.Gel8.pdf]

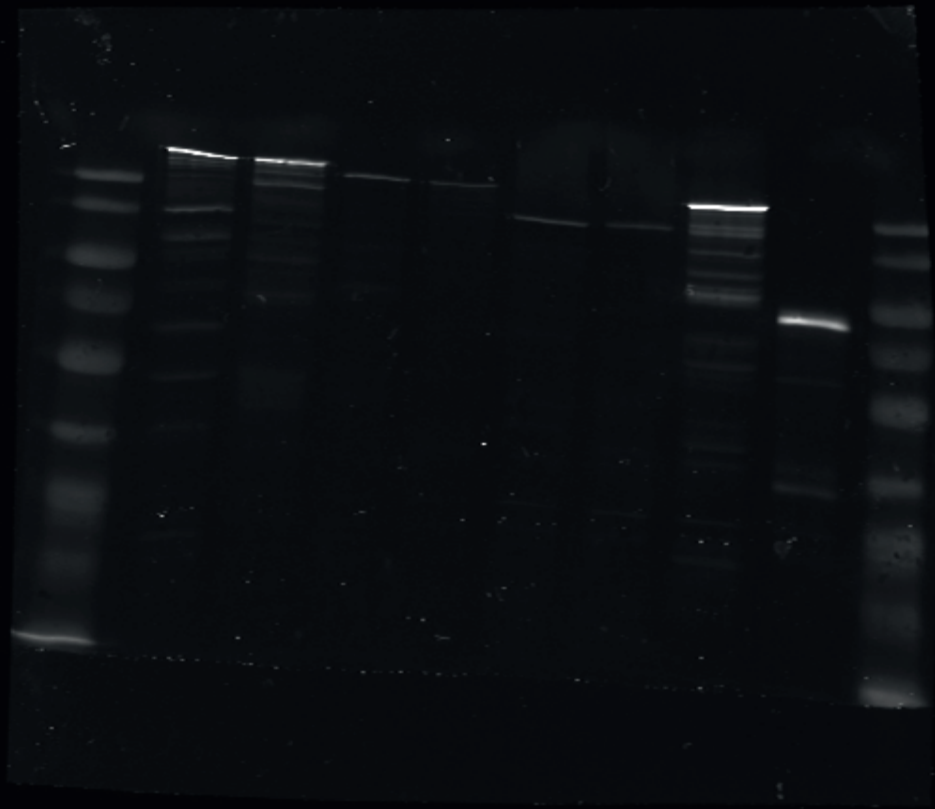

Supplement: Figure 5—figure supplement 1—source data 2. [file elife-95028-fig5-figsupp1-data2.zip › eLife.95028.SourceData2.Raw and unedited_gels_for_Figure5_FigureSupplement1/Figure 5ΓÇôfigure supplement 1-source data 2. Uncropped and Unabeled gels for Figure 5.Gel2.pdf]

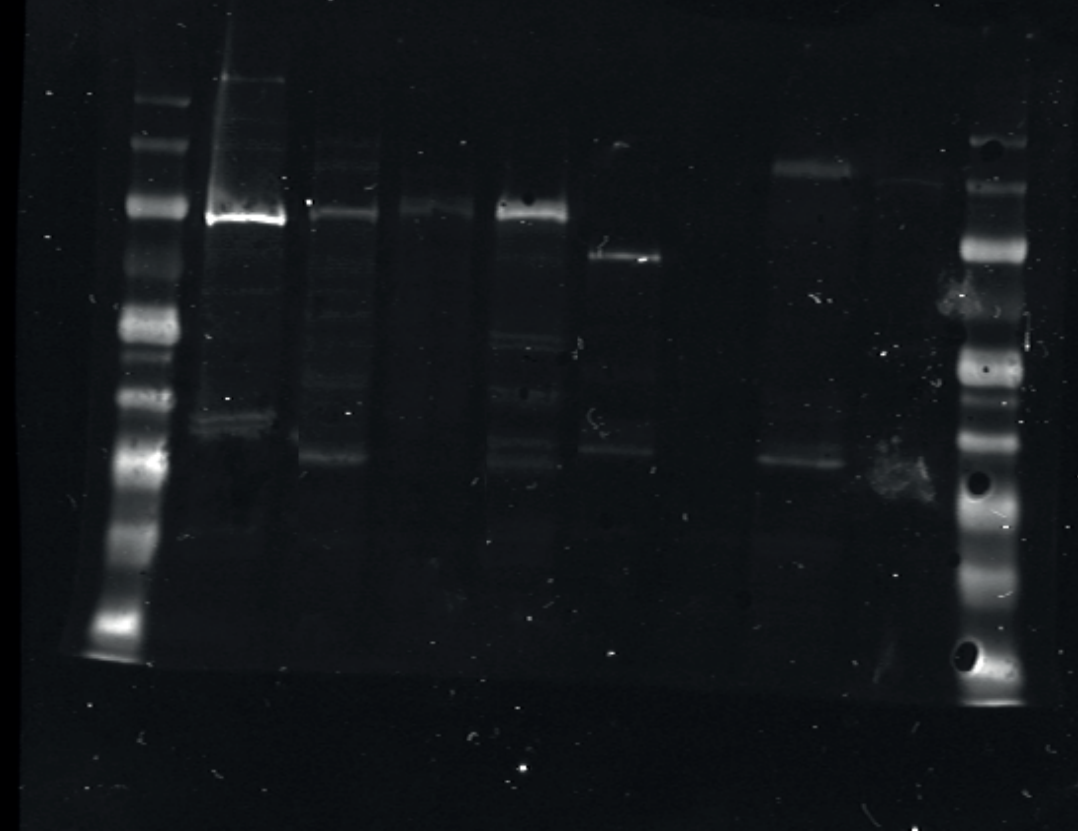

Supplement: Figure 5—figure supplement 1—source data 2. [file elife-95028-fig5-figsupp1-data2.zip › eLife.95028.SourceData2.Raw and unedited_gels_for_Figure5_FigureSupplement1/Figure 5ΓÇôfigure supplement 1-source data 2. Uncropped and Unabeled gels for Figure 5.Gel3.pdf]

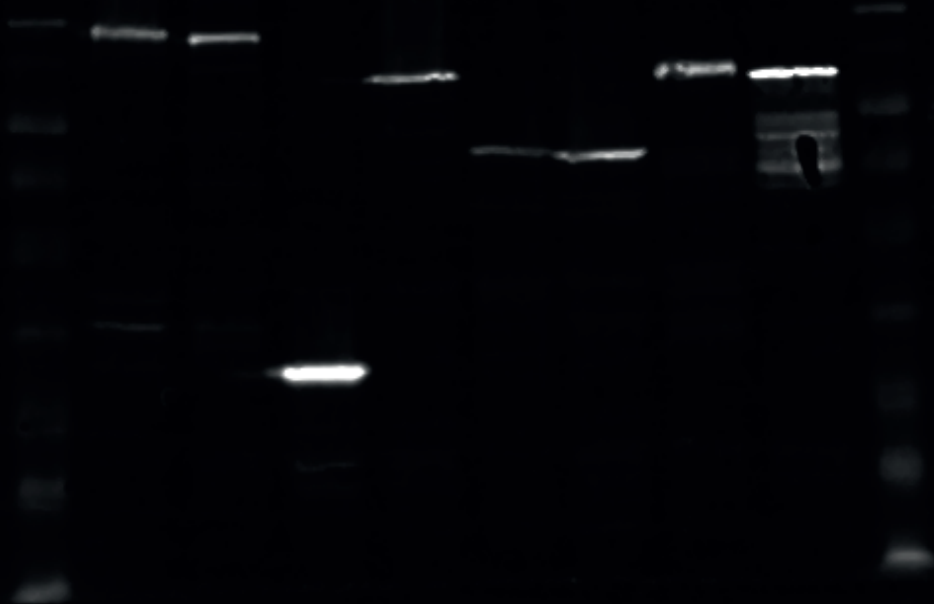

Supplement: Figure 5—figure supplement 1—source data 2. [file elife-95028-fig5-figsupp1-data2.zip › eLife.95028.SourceData2.Raw and unedited_gels_for_Figure5_FigureSupplement1/Figure 5ΓÇôfigure supplement 1-source data 2. Uncropped and Unlabeled gels for Figure 5.Gel11.pdf]

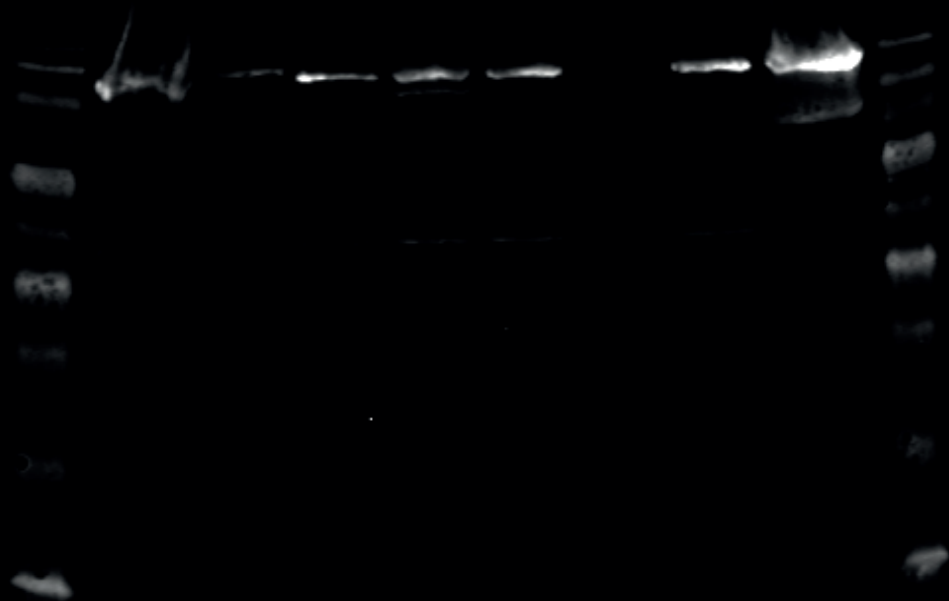

Supplement: Figure 5—figure supplement 1—source data 2. [file elife-95028-fig5-figsupp1-data2.zip › eLife.95028.SourceData2.Raw and unedited_gels_for_Figure5_FigureSupplement1/Figure 5ΓÇôfigure supplement 1-source data 2. Uncropped and Unlabeled gels for Figure 5.Gel10.pdf]

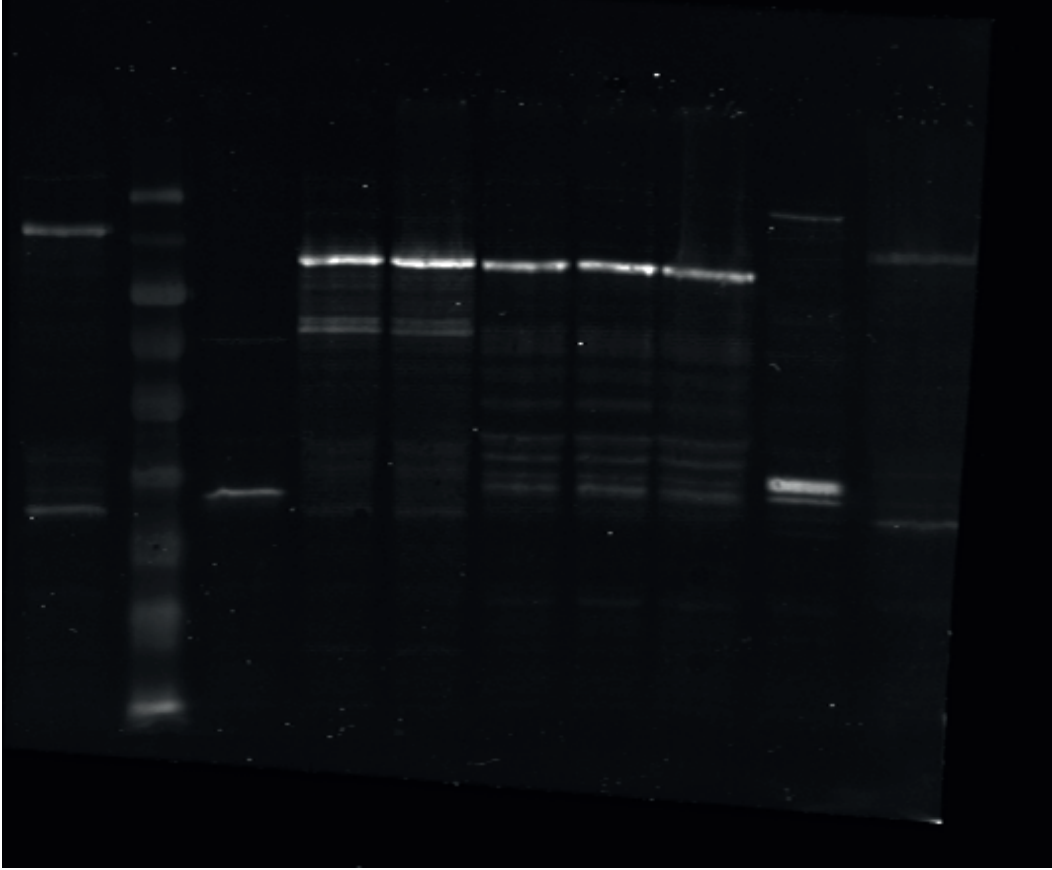

Supplement: Figure 5—figure supplement 1—source data 2. [file elife-95028-fig5-figsupp1-data2.zip › eLife.95028.SourceData2.Raw and unedited_gels_for_Figure5_FigureSupplement1/Figure 5ΓÇôfigure supplement 1-source data 2. Uncropped and Unlabeled gels for Figure 5.Gel4.pdf]

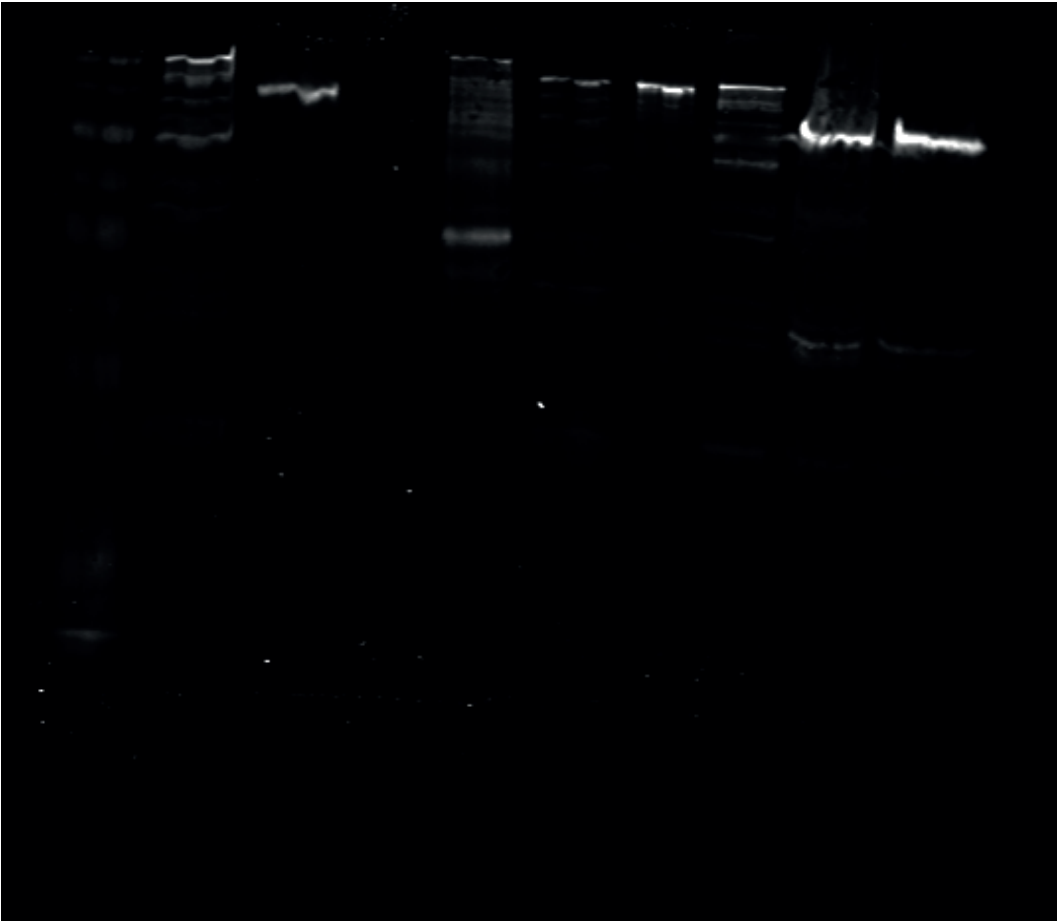

Supplement: Figure 5—figure supplement 1—source data 2. [file elife-95028-fig5-figsupp1-data2.zip › eLife.95028.SourceData2.Raw and unedited_gels_for_Figure5_FigureSupplement1/Figure 5ΓÇôfigure supplement 1-source data 2. Uncropped and Unlabeled gels for Figure 5.Gel5.pdf]

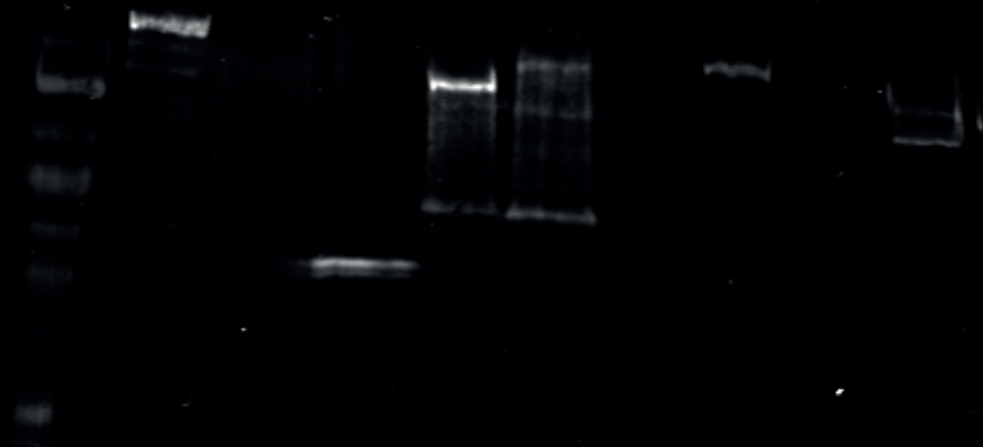

Supplement: Figure 5—figure supplement 1—source data 2. [file elife-95028-fig5-figsupp1-data2.zip › eLife.95028.SourceData2.Raw and unedited_gels_for_Figure5_FigureSupplement1/Figure 5ΓÇôfigure supplement 1-source data 2. Uncropped and Unlabeled gels for Figure 5.Gel7.pdf]

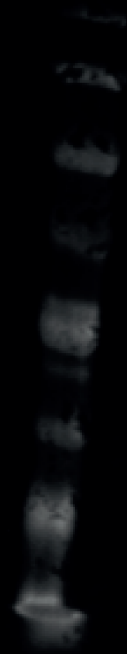

1

2

3

4

5

6

7

8

9

10

11

12

13

14

15

16

17

18

19

20

21

22

23

24

25

26

27

28

29

30

31

32

33

34

35

36

37

38

39

40

41

42

43

44

45

46

47

48

49

50

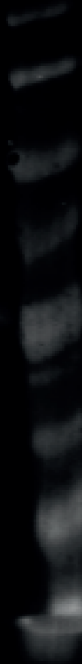

Supplement: Figure 5—figure supplement 1—source data 2. [file elife-95028-fig5-figsupp1-data2.zip › eLife.95028.SourceData2.Raw and unedited_gels_for_Figure5_FigureSupplement1/Figure 5ΓÇôfigure supplement 1-source data 2. Uncropped and Unlabeled gels for Figure 5.Gel6.pdf]

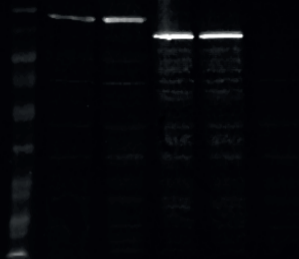

Supplement: Figure 5—figure supplement 1—source data 2. [file elife-95028-fig5-figsupp1-data2.zip › eLife.95028.SourceData2.Raw and unedited_gels_for_Figure5_FigureSupplement1/Figure 5ΓÇôfigure supplement 1-source data 2. Uncropped and Unlabeled gels for Figure 5.Gel1.pdf]
