# Supplementary material for: Detection of TurboID fusion proteins by fluorescent streptavidin outcompetes antibody signals and visualises targets not accessible to antibodies
Source: eLife. 2024 Aug 29;13:RP95028. doi: 10.7554/eLife.95028 (PMC11361705; doi:10.7554/eLife.95028)

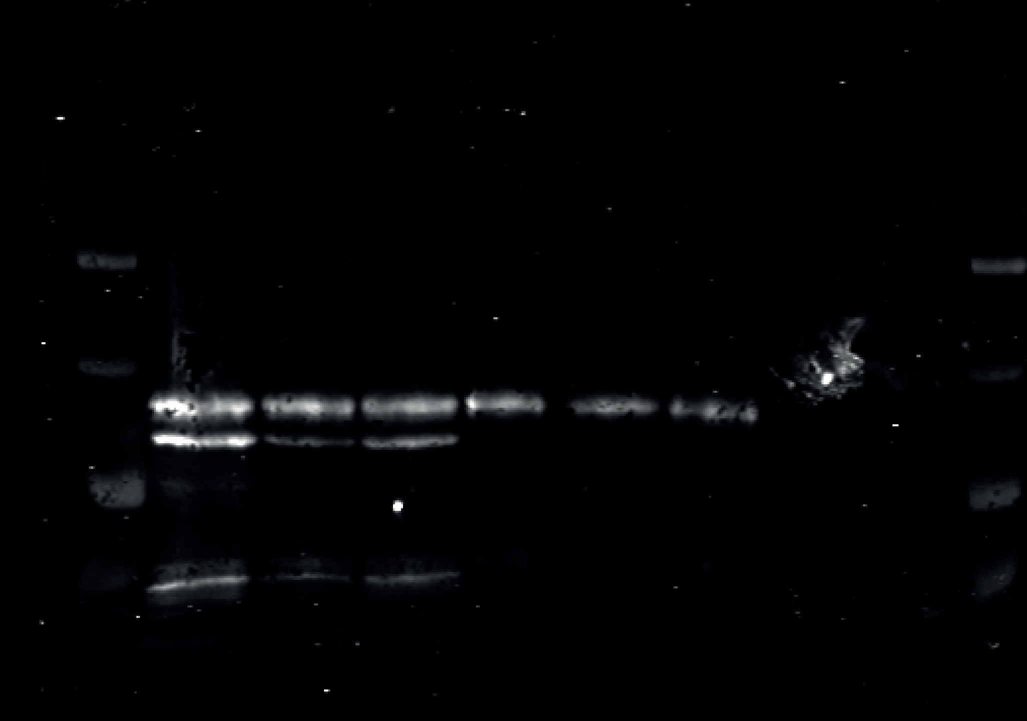

Supplement: Figure 5—figure supplement 1—source data 2. [file elife-95028-fig5-figsupp1-data2.zip › eLife.95028.SourceData2.Raw and unedited_gels_for_Figure5_FigureSupplement1/Figure 5ΓÇôfigure supplement 1-source data 2. Uncropped and Unlabeled gels for Figure 5.Gel9.pdf]
