# Supplementary material for: Detection of TurboID fusion proteins by fluorescent streptavidin outcompetes antibody signals and visualises targets not accessible to antibodies
Source: eLife. 2024 Aug 29;13:RP95028. doi: 10.7554/eLife.95028 (PMC11361705; doi:10.7554/eLife.95028)
Supplement: Supplementary file 1. [file elife-95028-supp1.docx]

**HeLa cells**

| ***protein*** | ***end tagged*** | ***tag*** | ***Lab-internal name*** | ***Sequence of the fusion protein*** | ***tagging method*** |
| --- | --- | --- | --- | --- | --- |
|  |  |  |  |  |  |
| NUP88 | N | TurboID-4HA | SK1196 | MKDNTVPLKLIALLANGEFHSGEQLGETLGMSRAAINKHIQTLRDWGVDVFTVPGKGYSLPEPIPLLNAKQILGQLDGGSVAVLPVVDSTNQYLLDRIGELKSGDACIAEYQQAGRGSRGRKWFSPFGANLYLSMFWRLKRGPAAIGLGPVIGIVMAEALRKLGADKVRVKWPNDLYLQDRKLAGILVELAGITGDAAQIVIGAGINVAMRRVEESVVNQGWITLQEAGINLDRNTLAATLIRELRAALELFEQEGLAPYLPRWEKLDNFINRPVKLIIGDKEIFGISRGIDKQGALLLEQDGVIKPWMGGEISLRSAEKASYPYDVPDYAYPYDVPDYAYPYDVPDYAYPYDVPDYAGSSGSDLELKLASMAAAEGPVGDGELWQTWLPNHVVFLRLREGLKNQSPTEAEKPASSSLPSSPPPQLLTRNVVFGLGGELFLWDGEDSSFLVVRLRGPSGGGEEPALSQYQRLLCINPPLFEIYQVLLSPTQHHVALIGIKGLMVLELPKRWGKNSEFEGGKSTVNCSTTPVAERFFTSSTSLTLKHAAWYPSEILDPHVVLLTSDNVIRIYSLREPQTPTNVIILSEAEEESLVLNKGRAYTASLGETAVAFDFGPLAAVPKTLFGQNGKDEVVAYPLYILYENGETFLTYISLLHSPGNIGKLLGPLPMHPAAEDNYGYDACAVLCLPCVPNILVIATESGMLYHCVVLEGEEEDDHTSEKSWDSRIDLIPSLYVFECVELELALKLASGEDDPFDSDFSCPVKLHRDPKCPSRYHCTHEAGVHSVGLTWIHKLHKFLGSDEEDKDSLQELSTEQKCFVEHILCTKPLPCRQPAPIRGFWIVPDILGPTMICITSTYECLIWPLLSTVHPASPPLLCTREDVEVAESPLRVLAETPDSFEKHIRSILQRSVANPAFLNCFTQLRKIKVETLFLRASEKDIAPPPEECLQLLSRATQVFREQYILKQDLAKEEIQRRVKLLCDQKKKQLEDLSYCREERKSLREMAERLADKYEEAKEKQEDIMNRMKKLLHSFHSELPVLSDSERDMKKELQLIPDQLRHLGNAIKQVTMKKDYQQQKMEKVLSLPKPTIILSAYQRKCIQSILKEEGEHIREMVKQINDIRNHVNFAS* | plasmid, pEGFP-N1 |
| NUP54 | N | TurboID-4HA | SK1197 | MKDNTVPLKLIALLANGEFHSGEQLGETLGMSRAAINKHIQTLRDWGVDVFTVPGKGYSLPEPIPLLNAKQILGQLDGGSVAVLPVVDSTNQYLLDRIGELKSGDACIAEYQQAGRGSRGRKWFSPFGANLYLSMFWRLKRGPAAIGLGPVIGIVMAEALRKLGADKVRVKWPNDLYLQDRKLAGILVELAGITGDAAQIVIGAGINVAMRRVEESVVNQGWITLQEAGINLDRNTLAATLIRELRAALELFEQEGLAPYLPRWEKLDNFINRPVKLIIGDKEIFGISRGIDKQGALLLEQDGVIKPWMGGEISLRSAEKASYPYDVPDYAYPYDVPDYAYPYDVPDYAYPYDVPDYAGSSGSDLELKLASMAFNFGAPSGTSGTAAATAAPAGGFGGFGTTSTTAGSAFSFSAPTNTGTTGLFGGTQNKGFGFGTGFGTTTGTSTGLGTGLGTGLGFGGFNTQQQQQTTLGGLFSQPTQAPTQSNQLINTASALSAPTLLGDERDAILAKWNQLQAFWGTGKGYFNNNIPPVEFTQENPFCRFKAVGYSCMPSNKDEDGLVVLVFNKKETEIRSQQQQLVESLHKVLGGNQTLTVNVEGTKTLPDDQTEVVIYVVERSPNGTSRRVPATTLYAHFEQANIKTQLQQLGVTLSMTRTELSPAQIKQLLQNPPAGVDPIIWEQAKVDNPDSEKLIPVPMVGFKELLRRLKVQDQMTKQHQTRLDIISEDISELQKNQTTSVAKIAQYKRKLMDLSHRTLQVLIKQEIQRKSGYAIQADEEQLRVQLDTIQGELNAPTQFKGRLNELMSQIRMQNHFGAVRSEERYYIDADLLREIKQHLKQQQEGLSHLISIIKDDLEDIKLVEHGLNETIHIRGGVFSAS* | plasmid, pEGFP-N1 |

***T. brucei***

| ***protein*** | ***GeneID number*** | ***end tagged*** | ***tag*** | ***Lab-internal name*** | ***tagging method*** | ***selection*** |
| --- | --- | --- | --- | --- | --- | --- |
|  |  |  |  |  |  |  |
| NUP64 | [Tb927.4.4310](https://tritrypdb.org/tritrypdb/app/record/gene/Tb927.4.4310) | N | TurboID-HA | SK953 | PCR | Puromycin |
| NUP64 | [Tb927.4.4310](https://tritrypdb.org/tritrypdb/app/record/gene/Tb927.4.4310) | C | TurboID-HA | SK954 | PCR | Puromycin |
| NUP149 | [Tb927.11.11080](https://tritrypdb.org/tritrypdb/app/record/gene/Tb927.11.11080) | N | TurboID-HA | SK955 | PCR | Puromycin |
| NUP149 | [Tb927.11.11080](https://tritrypdb.org/tritrypdb/app/record/gene/Tb927.11.11080) | C | TurboID-HA | SK956 | PCR | Puromycin |
| NUP89 | [Tb927.11.2950](https://tritrypdb.org/tritrypdb/app/record/gene/Tb927.11.2950) | N | TurboID-HA | SK957 | PCR | Puromycin |
| NUP89 | [Tb927.11.2950](https://tritrypdb.org/tritrypdb/app/record/gene/Tb927.11.2950) | C | TurboID-HA | SK958 | PCR | Puromycin |
| NUP75 | [Tb927.8.8050](https://tritrypdb.org/tritrypdb/app/record/gene/Tb927.8.8050) | N | TurboID-HA | SK959 | PCR | Puromycin |
| NUP75 | [Tb927.8.8050](https://tritrypdb.org/tritrypdb/app/record/gene/Tb927.8.8050) | C | TurboID-HA | SK960 | PCR | Puromycin |
| NUP98 | [Tb927.3.3180](https://tritrypdb.org/tritrypdb/app/record/gene/Tb927.3.3180) | N | TurboID-HA | SK961 | PCR | Puromycin |
| NUP98 | [Tb927.3.3180](https://tritrypdb.org/tritrypdb/app/record/gene/Tb927.3.3180) | C | TurboID-HA | SK962 | PCR | Puromycin |
| NUP53a | [Tb927.11.15560](https://tritrypdb.org/tritrypdb/app/record/gene/Tb927.11.15560) | N | TurboID-HA | SK963 | PCR | Puromycin |
| NUP53a | [Tb927.11.15560](https://tritrypdb.org/tritrypdb/app/record/gene/Tb927.11.15560) | C | TurboID-HA | SK964 | PCR | Puromycin |
| NUP53b | [Tb927.3.3540](https://tritrypdb.org/tritrypdb/app/record/gene/Tb927.3.3540) | N | TurboID-HA | SK965 | PCR | Puromycin |
| NUP53b | [Tb927.3.3540](https://tritrypdb.org/tritrypdb/app/record/gene/Tb927.3.3540) | C | TurboID-HA | SK966 | PCR | Puromycin |
| NUP62 | [Tb927.4.5200](https://tritrypdb.org/tritrypdb/app/record/gene/Tb927.4.5200) | N | TurboID-HA | SK967 | PCR | Puromycin |
| NUP62 | [Tb927.4.5200](https://tritrypdb.org/tritrypdb/app/record/gene/Tb927.4.5200) | C | TurboID-HA | SK968 | PCR | Puromycin |
| NUP109 | [Tb927.11.15990](https://tritrypdb.org/tritrypdb/app/record/gene/Tb927.11.15990) | N | TurboID-HA | SK969 | PCR | Puromycin |
| NUP109 | [Tb927.11.15990](https://tritrypdb.org/tritrypdb/app/record/gene/Tb927.11.15990) | C | TurboID-HA | SK970 | PCR | Puromycin |
| NUP82 | [Tb927.9.14240](https://tritrypdb.org/tritrypdb/app/record/gene/Tb927.9.14240) | C | TurboID-HA | SK972 | PCR | Puromycin |
| NUP41 | [Tb927.10.2320](https://tritrypdb.org/tritrypdb/app/record/gene/Tb927.10.2320) | N | TurboID-HA | SK973 | PCR | Puromycin |
| NUP41 | [Tb927.10.2320](https://tritrypdb.org/tritrypdb/app/record/gene/Tb927.10.2320) | C | TurboID-HA | SK974 | PCR | Puromycin |
| Sec13 | [Tb927.10.14180](https://tritrypdb.org/tritrypdb/app/record/gene/Tb927.10.14180) | N | TurboID-HA | SK975 | PCR | Puromycin |
| NUP132 | [Tb927.7.2300](https://tritrypdb.org/tritrypdb/app/record/gene/Tb927.7.2300) | N | TurboID-HA | SK977 | PCR | Puromycin |
| NUP132 | [Tb927.7.2300](https://tritrypdb.org/tritrypdb/app/record/gene/Tb927.7.2300) | C | TurboID-HA | SK978 | PCR | Puromycin |
| NUP152 | [Tb927.10.9650](https://tritrypdb.org/tritrypdb/app/record/gene/Tb927.10.9650) | N | TurboID-HA | SK979 | PCR | Puromycin |
| NUP152 | [Tb927.10.9650](https://tritrypdb.org/tritrypdb/app/record/gene/Tb927.10.9650) | C | TurboID-HA | SK980 | PCR | Puromycin |
| NUP119 | [Tb927.11.9780](https://tritrypdb.org/tritrypdb/app/record/gene/Tb927.11.9780) | N | TurboID-HA | SK981 | PCR | Puromycin |
| NUP119 | [Tb927.11.9780](https://tritrypdb.org/tritrypdb/app/record/gene/Tb927.11.9780) | C | TurboID-HA | SK982 | PCR | Puromycin |
| NUP181 | [Tb927.10.8910](https://tritrypdb.org/tritrypdb/app/record/gene/Tb927.10.8910) | N | TurboID-HA | SK983 | PCR | Puromycin |
| NUP181 | [Tb927.10.8910](https://tritrypdb.org/tritrypdb/app/record/gene/Tb927.10.8910) | C | TurboID-HA | SK984 | PCR | Puromycin |
| NUP225 | [Tb927.4.2880](https://tritrypdb.org/tritrypdb/app/record/gene/Tb927.4.2880) | N | TurboID-HA | SK985 | PCR | Puromycin |
| NUP225 | [Tb927.4.2880](https://tritrypdb.org/tritrypdb/app/record/gene/Tb927.4.2880) | C | TurboID-HA | SK986 | PCR | Puromycin |
| NUP144 | [Tb927.10.8170](https://tritrypdb.org/tritrypdb/app/record/gene/Tb927.10.8170) | N | TurboID-HA | SK987 | PCR | Puromycin |
| NUP144 | [Tb927.10.8170](https://tritrypdb.org/tritrypdb/app/record/gene/Tb927.10.8170) | C | TurboID-HA | SK988 | PCR | Puromycin |
| NUP65 | [Tb927.10.3810](https://tritrypdb.org/tritrypdb/app/record/gene/Tb927.10.3810) | N | TurboID-HA | SK989 | PCR | Puromycin |
| NUP65 | [Tb927.10.3810](https://tritrypdb.org/tritrypdb/app/record/gene/Tb927.10.3810) | C | TurboID-HA | SK990 | PCR | Puromycin |
| NUP92 | [Tb927.9.1340](https://tritrypdb.org/tritrypdb/app/record/gene/Tb927.9.1340) | N | TurboID-HA | SK991 | PCR | Puromycin |
| NUP92/MLP2 | [Tb927.9.1340](https://tritrypdb.org/tritrypdb/app/record/gene/Tb927.9.1340) | C | TurboID-HA | SK992 | PCR | Puromycin |
| NUP96 | [Tb927.10.7060](https://tritrypdb.org/tritrypdb/app/record/gene/Tb927.10.7060) | N | TurboID-HA | SK925 | PCR | Puromycin |
| NUP96 | [Tb927.10.7060](https://tritrypdb.org/tritrypdb/app/record/gene/Tb927.10.7060) | C | TurboID-HA | SK899 | PCR | Puromycin |
| NUP110 | [Tb927.11.330](https://tritrypdb.org/tritrypdb/app/record/gene/Tb927.11.330) | N | TurboID-HA | SK924 | PCR | Puromycin |
| NUP110 | [Tb927.11.330](https://tritrypdb.org/tritrypdb/app/record/gene/Tb927.11.330) | C | TurboID-HA | SK897 | PCR | Puromycin |
| NUP76 | [Tb927.8.6250](https://tritrypdb.org/tritrypdb/app/record/gene/Tb927.8.6250) | N | TurboID-HA | SK926 | PCR | Puromycin |
| NUP76 | [Tb927.8.6250](https://tritrypdb.org/tritrypdb/app/record/gene/Tb927.8.6250) | C | TurboID-HA | SK896 | PCR | Puromycin |
| NUP158 | [Tb927.11.980](https://tritrypdb.org/tritrypdb/app/record/gene/Tb927.11.980) | N | TurboID-HA | SK922 | PCR | Puromycin |
| NUP158 | [Tb927.11.980](https://tritrypdb.org/tritrypdb/app/record/gene/Tb927.11.980) | C | TurboID-HA | SK550 | plasmid (SK527) | neomycin |
| NUP140 | [Tb927.11.11090](https://tritrypdb.org/tritrypdb/app/record/gene/Tb927.11.11090) | N | TurboID-HA | SK923 | PCR | Puromycin |
| MEX67 | [Tb927.11.2370](https://tritrypdb.org/tritrypdb/app/record/gene/Tb927.11.2370) | C | TurboID-HA | SK560 | plasmid (SK527) | Neomycin |
| MEX67 | [Tb927.11.2370](https://tritrypdb.org/tritrypdb/app/record/gene/Tb927.11.2370) | C | eGFP | SK1099 | PCR | Puromycin |
| MEX67 | [Tb927.11.2370](https://tritrypdb.org/tritrypdb/app/record/gene/Tb927.11.2370) | C | TurboID-1Ty1 | SK1060 | PCR | Puromycin |
| NOG1 | [Tb927.11.3120](https://tritrypdb.org/tritrypdb/app/record/gene/Tb927.11.3120) | C | TurboID-HA | SK920 | PCR | Puromycin |
| NOG1 | [Tb927.11.3120](https://tritrypdb.org/tritrypdb/app/record/gene/Tb927.11.3120) | N | eGFP | SK1108 | PCR | Puromycin |
| NOG1 | [Tb927.11.3120](https://tritrypdb.org/tritrypdb/app/record/gene/Tb927.11.3120) | N | TurboID-1Ty1 | SK1109 | PCR | Puromycin |
| PABP2 | [Tb927.9.10770](https://tritrypdb.org/tritrypdb/app/record/gene/Tb927.9.10770) | C | TurboID-HA | SK538 | plasmid (SK527) | Neomycin |
| PABP2 | [Tb927.9.10770](https://tritrypdb.org/tritrypdb/app/record/gene/Tb927.9.10770) | C | mChFP | 3342 | plasmid (3086) | Hygromycin |
| ALPH1 | [Tb927.6.640](https://tritrypdb.org/tritrypdb/app/record/gene/Tb927.6.640) | C | eYFP | SK336 | plasmid (3888), tetracyclin inducible | Blasticidin |
| PABP2 | [Tb927.9.10770](https://tritrypdb.org/tritrypdb/app/record/gene/Tb927.9.10770) | C | eYFP | 3295 | plasmid | Neomycin |
